# Book Reviews

**Published:** 1875-10

**Authors:** 


### Book Reviews.

[NOTE. — All works reviewed in the pages of the CHICAGO MEDICAL JOURNAL AND EXAMINER may be found in the extensive stock of W. B. KEEN, COOKE & CO., whose catalogue of Medical Books will be sent to any address upon request.]

ON PARALYSIS FROM BRAIN DISEASE IN ITS COMMON FORMS. By *H. Charlton Bastian, M.D., Etc.* New York: D. Appleton & Co. 1875. pp. 335. Price, $1.75.

It is as pleasurable as it is rare, among the multitude of books that appear to have been born because their authors and not the world were in need of them, to come to one that is valuable throughout and that has hardly a defect. Yet such is the volume before us. It comprises eight lectures delivered on this interesting subject by Dr. Bastian in University College Hospital in 1874. Previous to their publication in book form the lectures were revised and somewhat enlarged.

The author discusses the subject from a purely clinical stand-point, in marked contrast with the manner heretofore generally in vogue among writers on this subject, and of which he complains, of considering the topic almost solely from the aspect of pathological science.

There is no objection to pathological studies ; these are valuable. But any one of several different kinds of lesion in a given part of the brain may cause the same group of symptoms ; so that a most important study of the subject, for the active physician especially, must be that which considers, from the symptoms and history, the location in the brain of the lesion, the extent and nature of the damage, and, as far as possible from such information, the pathological nature of the lesion in each case, the prognosis and treatment. Such a study the author attempts to supply, while at the same time a tolerably systematic consideration of the whole subject is presented. As he truly says of his subject, "No department of medicine stands more in need of being represented in a text book of moderate compass.''

In the first lecture the causes of hemiplegia are briefly considered, ''with the view of obtaining data available for purposes of diagnosis.'' The only causes thoroughly discussed, however, with their more immediate consequences, are rupture, occlusion and spasm of the cerebral arteries. Meningeal, cerebral and cerebellar hæmorrhage, thrombosis and embolism, and the softening often so rapidly following these, are considered at considerable length, while induration, tumors, abscesses, injuries and congenital atrophies are barely mentioned, as they relate to the more slowly arising and more irregular forms of hemiplegia. About spasm of vessels as a cause, it is very safely said, ''there is a great deal of uncertainty.'' There is probably an altered molecular state in the brain tissue which accounts for the spasms which come and go so quietly that they leave no organic change by which we may be *sure* they ever existed, and yet the author has no doubt many cases of hemiplegia are to be placed in this category. Here he groups many cases of ''Epileptic Hemiplegia,'' ''Emotional Hemiplegia,'' and a few ''cases of an hysterical character.'' Patients with various symptoms that class them under these respective heads are attacked, fall down, become hemiplegic and after a variable time and possibly many vicissitudes recover, and no discoverable lesion tells us positively of the condition that existed. Spasm of vessels is the explanation offered ; but sparseness of facts that can be presented bearing on this point make it one of great interest in clinical study.

Speaking of hemichorea as being followed by hemiplegia of the same side, the author believes in a certain number of such cases ''minute vessels become blocked in the corpus striatum and adjacent parts of the brain,'' he himself having found this on three or four occasions.

In the second lecture hereditary influences as favoring hemiplegia are considered; then follows an admirably concise statement of the important knowledge—that from both early and recent investigations—concerning the distribution and relations of the cerebral blood vessels, the lecture closing with a discussion in part of the symtomatology of hemiplegia, which is continued through succeeding lectures.

A lecture is devoted to alteration of nutrition, degeneration of the cord, and the symptoms due to the functional differences between the two cerebral hemispheres; another to regional diagnosis in brain disease—the most valuable chapter in the book, in our estimation; the seventh considers the cerebellum, its functions and symptomatology, and closes with half a dozen pages on "pathological diagnosis," while in the closing lecture the subjects of prognosis and treatment are briefly considered.

With Broadbent, the author denies for the optici thalami the seat of the 'sense centre' for common sensation, but locates it on the upper and posterior part of the pons Varolii, thereby agreeing with "many eminent physiologists."

Of the author's recorded cases of hemiplegia about three and one-half per cent. were of the irregular and exceptional form in which the upper extremity recovers more rapidly than the lower—the reverse being the rule. He does not think these causes are necessarily attended with dementia or an earlier fatality than the more common forms—herein differing with the opinion of Trousseau.

After referring at considerable length to aphasia and other conditions and symptoms due to a lesion in the left hemisphere, we are told that lesions of the right hemisphere are more often fatal than those of the left; that, too, disorders of nutrition on the paralyzed side and convulsion and spasms of these parts, as well as the "conjugated deviation of the eyes," "hysterical paralysis," and paralysis and convulsions of muscles on the same side of the body with the lesion, occur most with lesion on the right side. "The occurrence of paralysis or of convulsions on the same side as the brain lesion, is, with our present knowledge, quite inexplicable." But the author suggests that it might be explained by supposing some vice of development had led to a failure in the perfect decussation of the fibres in the motor tract, and thinks it better to entertain the possibility of such an event than to distrust the regularity of the phenomena produced by brain lesion.

He says the habit of bleeding and purging is now so completely abandoned that it is needless to object to it—in which statement he is in some degree mistaken, at least so far as this country is concerned.

He advises strongly against the early use of faradization of the muscles, and condemns the sending of galvanic currents through the head at any stage. He is not aware of any sound principle upon which such haphazard attempts can be commended. In the rare cases of the early wasting of muscles "much good may be done by faradization." With this brief reference he dismisses the subject of electricity.

It is to be regretted that more of the author's sensible notions on treatment are not given. Yet this defect we can hardly complain of as the major promise was to discuss clinical considerations, and as upwards of 200 pages are devoted to a review of symptomatology and original diagnosis.

What the lectures omit, rather than any statements they contain, will be a disappointment to the reader. The treatment of the subject is so admirable that one instinctively finds himself wishing more had been said on some points, where space and time seem to have compelled the author to abbreviate. Altogether the book is an excellent one. The profession can well bear to be given more books of this kind; they are needed. We need more monographs generally, and we can particularly profit by more books like this which amounts to a digest of the best studies on the subject it treats, condensed into a small space by an able hand, with his own studies and opinions to tone the whole.

CLINICAL LECTURES AND ESSAYS. By *Sir James Paget, F.S.R.* Edited by Howard Marsh, F.R.C.S. New York: D. Appleton & Co. 1875. Price, $5.00.

Although most of these lectures have already been published in various medical journals, the profession is greatly indebted to Mr. Marsh for having collected them into one volume. Paget's writings are of more than a transient interest, and this book is a valuable contribution to medical literature. We admire the rich experience and keen observation as well as the great sincerity of the eminent surgeon. It is a pleasure indeed to read a book which, on every page, leaves the stamp of truthfulness and wise judgment. Distinguished by his operative skill he does not underrate the minor parts of surgery in their influence upon the ultimate success of operations; speaking of the calamities of surgery he graphically demonstrates the great importance of carefully dressing the wounds and looking to all the seemingly little things that, after an operation, minister to a patient's comfort and welfare. And he also gives the sound advice that before deciding upon an operation, "you should examine the patient with at least as much care as you would for a life-insurance." But we need not enter into the details of the contents of a book every physician ought to read in full. F. C. H.

THE MULTUM IN PARVO REFERENCE AND DOSE BOOK. By *C. Henri Leonard, M.A., M.D.* Second Edition. Detroit. Paper, 60 cts.; cloth, 75 cts.

Table of contents contains—List of Doses, Officinal Preparations, Rules of Pronunciation and of Genitive-Case Endings, Incompatibles, Poisons, Antidotes and Tests, Urinalysis, Obstetric Syllabi, Visceral Measurements, Epitomizations of the Exanthemata, Pronunciation of Medico-Biographical Names, Ethics, Fee-bill and Emergency Expediencies. Mindful of, and appreciating the thousand-and-one "Casebooks" and "Handbooks" for Physicians, still every one must acknowledge that this author has compressed an immense amount of ready, practical information into the minimum space.

### BOOKS RECEIVED.

THE TEST POCKET ANATOMIST, (founded on Gray). By *C. Henri Leonard, A.M., M.D.* Detroit. 50 cts.

### PAMPHLETS RECEIVED.

RELATIONS OF THE NERVOUS SYSTEM TO DISEASES OF THE SKIN. By *L. D. Bulkley, A.M., M.D.*

TRANSACTIONS OF MEDICAL ASSOCIATION OF ALABAMA.

AMERICAN CLINICAL LECTURES: Capillary Bronchitis of Adults. By *Prof. Calvin Ellis, M.D.*

FRACTURES OF THE INFERIOR MAXILLARY JAW. By *Dr. J. F. Montgomery*, Sacramento, Cal.

### EXCHANGES.

Cincinnati Lancet and Observer—September.
New York Med. Record—Aug. 28, Sept. 4, 11.
Philadelphia Med. Times—Aug. 28, Sept. 4, 11, 18.
The Clinic—Aug. 28, Sept. 4, 11, 18.
Richmond and Louisville Med. Journal—August.
The Practitioner—July and August.
Canada Lancet—July.
Boston Med. and Surg. Journal—Aug. 26, Sept. 2, 9, 16.
Students' Jour. and Hospital Gazette—Aug. 14, 28.
Chemist and Druggist.
Med. and Surg. Reporter—Aug. 28, Sept. 4, 11.
Centralblatt f. Chirurgie, N. 31, 32, 33, 34, 35, 36.
New York Med. Journal—Sept.
Nashville Med. Journal—Sept.
American Med. Weekly—Aug. 28, Sept. 4, 11.
Atlanta Med. and Surg. Journal—Sept.
Allgemeine Medicinische Central Zeitung—62, 63, 64, 65, 66.
Allgemeine Wiener Med. Zeitung, 31, 32, 33, 34, 35.
Photographic News—August.
Boston Journal of Chemistry—Sept.
Virginia Med. Monthly—Sept.
The Druggists' Circular—Sept.
Nashville Journal of Med. and Surg.—Sept.

The Med. Herald—Aug.
The Pharmacist—Sept.
Detroit Review of Medicine—Aug. and Sept.
New Orleans Med. and Surg. Jour.—Sept.
St. Louis Clinical Record—Sept.
Tho Doctor—Sept.
The Dental Cosmos—Sept.
Am. Dental Journal—Sept.
Progres Médicale—July 28, Aug. 7, 14, 21, 28.
Memorabilien—Aug. 26.
Irrenfreund, No. 6.
Pacific Med. and Surg. Journal—Sept.
London Lancet—Sept.
La France Médicale—Aug. 25, 28.

